# Comparison of Stress-Hemoconcentration Correction Techniques for Stress-Induced Coagulation

**DOI:** 10.1155/2013/480648

**Published:** 2013-10-07

**Authors:** Anthony W. Austin, Stephen M. Patterson

**Affiliations:** ^1^Montreal Behavioural Medicine Centre, Hôpital du Sacré-Coeur de Montréal, Office J-3145, 5400 Boulevard Gouin Ouest, Montreal, Quebec, Canada H4J 1C5; ^2^Department of Exercise Science, Concordia University, Montreal, Quebec, Canada H4B 1R6; ^3^Research Centre, Hôpital du Sacré-Coeur de Montréal, Montreal, Quebec, Canada H4J 1C5; ^4^Department of Psychology, Ohio University, 200 Porter Hall, Athens, OH 45701, USA

## Abstract

When examining stress effects on coagulation, arithmetic correction is typically used to adjust for concomitant hemoconcentration but may be inappropriate for coagulation activity assays. We examined a new physiologically relevant method of correcting for stress-hemoconcentration. Blood was drawn from healthy men (*N* = 40) during baseline, mental stress, and recovery, and factor VII activity (FVII:C), factor VIII activity (FVIII:C), activated partial thromboplastin time (APTT), prothrombin time (PT%), fibrinogen, D-dimer, and plasma volume were determined. Three hemoconcentration correction techniques were assessed: arithmetic correction and two reconstitution techniques using baseline plasma or physiological saline. Area-under-the-curve (AUC) was computed for each technique. For FVII:C, uncorrected AUC was significantly greater than AUC corrected arithmetically. For PT%, uncorrected AUC was significantly greater than AUC corrected with saline or arithmetically. For APTT, uncorrected AUC was significantly less than AUC corrected with saline and greater than AUC corrected arithmetically. For fibrinogen, uncorrected AUC was significantly greater than AUC corrected with saline or arithmetically. For D-dimer, uncorrected AUC was significantly greater than AUC corrected arithmetically. No differences in AUC were observed for FVIII:C. Saline reconstitution seems most appropriate when adjusting for hemoconcentration effects on clotting time and activity. Stress-hemoconcentration accounted for the majority of coagulation changes.

## 1. Introduction

Coronary heart disease (CHD) is the primary cause of death in the United States [1]. Conventional risk factors, such as hypertension, hypercholesterolemia, diabetes, and smoking, do not fully explain CHD risk [2]. Psychosocial factors, such as stress and depression, may explain additional risk. For example, the INTERHEART epidemiological study [3] suggests that individuals who had suffered from myocardial infarction reported more financial stress, more stress at home and work, and more major life events in the year prior to their infarction compared to controls. Moreover, acute psychosocial stress, such as that arising from taxing social situations or intense negative emotions, may trigger atherothrombotic events, especially myocardial infarction [4]. Many pathophysiological processes may explain the link between stress and CHD, including hemoconcentration [5, 6], increased blood viscosity [7, 8], and elevated platelet and coagulation activation [9, 10], all factors that intensify shear stress on atherosclerotic plaques, thereby promoting plaque ruptures.

A large body of hemostasis research suggests that acute psychosocial stress concurrently activates coagulation and fibrinolysis, but the former increases to a greater extent than the latter, resulting in net hypercoagulability [11]. Especially among those with CHD, such a physiological response could put one at increased risk for coronary thrombosis after plaque disturbance and subsequently contribute to acute coronary syndromes [11–13]. The mechanistic underpinnings of acute stress-induced hypercoagulation are incompletely understood but probably involve hemoconcentration [14, 15]. In healthy individuals, an increase in blood pressure during acute psychological stress forces intravascular fluid (plasma filtrate) through capillary pores into interstitial spaces, whereas high-molecular weight molecules (i.e., greater than 69 kDa) cannot pass through, thereby increasing the concentration of such large molecules [5]. Notably, most clotting factors are larger than 69 kDa. Despite a very large number of studies examining the influence of stress on coagulation, only a few have taken into account hemoconcentration effects [14–17]. Two of the earlier studies [16, 17] suggested that stress-induced changes in coagulation markers are not resultant from hemoconcentration. However, statistical techniques less statistically powerful than arithmetic adjustment were used for plasma volume (PV) shifts [18]. More recently, von Känel et al. [15] arithmetically corrected for hemoconcentration induced by a laboratory psychological stress task, using a widely accepted formula [19]. Arithmetic adjustment for PV shifts did not alter FVIII clotting activity (FVIII:C), FXII clotting activity (FXII:C), von Willebrand factor antigen, or D-dimer concentration. However, relative to baseline, arithmetic adjustment brought about a significant decrease in fibrinogen, FVII:C, activated partial thromboplastin (APTT), and prothrombin time activity (PT%). The adjusted result for APTT is puzzling, because a decrease in fibrinogen would be expected to be associated with a prolongation in APTT. To put it simply, a reduction in a clotting factor should be related to a decelerated clotting response. Moreover, and perhaps more importantly, arithmetic adjustment is meant to mathematically adjust the concentration of a molecule and has no time component. Thus, it is problematic to use such arithmetic adjustment for clotting time and clotting activity.

A more biologically suitable method of correcting for hemoconcentration when examining clotting time and clotting activity may be to physically correct for PV changes by reconstituting plasma samples collected during an acute stressor with a relevant fluid (i.e., physiological saline or the person's own plasma from rest) such that PV is returned to baseline levels [14]. Saline is characteristic of the filtrate lost during acute stress which consists predominantly of water and electrolytes. The efficacy of this technique is informed by the process of performing many clotting assays, in which plasma is diluted prior to analysis. For example, progressive dilutions with saline from 0% to 90% increased APTT and PT in seconds and reduced fibrinogen concentration and FVII:C exponentially [20]. However, only one study has showed that saline reconstitution is an appropriate method of adjusting for hemoconcentration [14]. On the other hand, reconstitution with the person's own plasma from a baseline period had no influence on clotting [14]. An important limitation of this study was the large number of post hoc tests conducted (i.e., 20 per parameter), increasing the risk of Type I error.

The present study further examined relationships between hemoconcentration and coagulation during acute stress. In addition to multiple post hoc tests, we compared area-under-the-curve [21] for each parameter after each hemoconcentration correction technique in order to minimize comparisons and reduce the potential for Type I error. Specifically, we hypothesized the following: (1) during stress, fibrinogen, D-dimer, PT%, and FVII:C that were uncorrected or corrected with plasma reconstitution would be greater than when corrected arithmetically or with saline reconstitution; (2) during stress, uncorrected APTT or APTT corrected with plasma reconstitution would be less than APTT corrected with saline reconstitution and greater than APTT corrected arithmetically; (3) during stress and recovery, FVIII:C corrected arithmetically would be less than uncorrected FVIII:C or FVIII:C corrected with saline or plasma reconstitution.

## 2. Methods

### 2.1. Participants

Sixty-four male university students over 18 years of age and enrolled in introductory psychology courses were screened for testing. Women were not examined in order to avoid difficulty in controlling for phase of the menstrual cycle. Exclusionary criteria were the following: (a) history of cardiovascular disease, thromboembolic event(s), or other chronic diseases; (b) use of aspirin, anti-inflammatories, antidepressants, or medications that affect blood pressure (e.g., beta blockers and diuretics); (c) smokers; (d) body mass index (BMI) greater than 30 kg/m^2^; (e) any current major or minor infection; (f) any trauma or surgery within the previous six months; (g) resting systolic blood pressure over 140 mm Hg or under 85 mm Hg, and diastolic blood pressure over 90 mm Hg or under 55 mm Hg. Participants were asked to refrain from exercising and from drinking alcohol for 24 hours prior to their session and to abstain from food and drink for four hours prior to their laboratory session but were allowed to drink water. Twenty individuals were excluded for the following reasons: 4 for BMI > than 30, 11 for eating within four hours prior to the study, one for exercising within 24 hours prior to the study, one for drinking alcohol within 24 hours prior to the study, one for history of fainting during blood draws/donation, one for high blood pressure, and one for having an acute infection. Four individuals did not complete the study due to a syncope reaction or lightheadedness during baseline period or did not want a catheter in their arm.  Table 1 contains the participant characteristics of the 40 participants who completed the study. This study was conducted with the understanding and consent of each participant, and it received ethics approval from the Biomedical Institutional Review Board at Ohio University.

### 2.2. Procedure

Upon laboratory arrival, informed consent was obtained and eligibility was verified. Next, to verify blood pressure, resting blood pressure was determined via the auscultatory method. To verify nonsmoking status, participants blew into a calibrated piCO Smokerlyzer (Bedfont Scientific Ltd., Kent, UK) and were excluded if the carbon monoxide result was greater than 10 parts per million. To verify no consumption of alcohol in the previous 24 hours, they blew into a calibrated AlcoHAWK PT500 Breath Alcohol Tester (Q3 Innovations, Independence, IA, USA) and were excluded if the blood alcohol content was greater than 0.00%. Height and weight were measured and BMI was calculated (BMI = kg/m^2^). Eligible participants then sat in a comfortable chair in a quiet room. An indwelling venous 20-gauge catheter (Exel Safelet Cath, Exelint International Co., Los Angeles, CA, USA) was inserted in a suitable vein in the antecubital fossa. A blood pressure cuff was placed on the opposite arm. Participants then rested quietly while listening to calm music during a 20-minute baseline period. Next, participants performed a 6-minute mental arithmetic task with strong verbal encouragement from the experimenter. Each participant was instructed to subtract aloud by sevens from a prerecorded 4-digit number as quickly and accurately as possible, with a new number presented each minute. The math stressor was followed by a 20-minute recovery period in which participants again rested quietly while listening to calm music. Blood samples for hemoconcentration and hemostasis measures were collected during the last minute of baseline, math, and recovery. Heart rate and blood pressure were assessed at minutes 15, 17, and 19 of baseline, minutes 0.5, 2.5, and 4.5 of math, and minutes 1, 3, 5, 7, 9, 11, 13, 15, 17, and 19 of recovery. Finally, the catheter and blood pressure cuff were removed.

### 2.3. Measures

#### 2.3.1. Hemodynamic Measures

A Colin Press-Mate BP-8800 automated blood pressure monitor (Colin Medical Instruments) was used to measure heart rate, SBP, and DBP at fixed intervals during baseline, math task, and recovery.

#### 2.3.2. Blood Sampling

At baseline, blood was collected into one 4 mL EDTA vacutainer tube for hemoconcentration measures and three 4.5 mL 3.2% sodium citrate tubes for coagulation measures. During the last minute of math and recovery, blood was collected into one 4 mL EDTA vacutainer tube for hemoconcentration measures and four 3.2% sodium citrate tubes for coagulation measures. Immediately after being obtained, the sodium citrate tubes were centrifuged for 10 minutes at 3000 ×g. Then, 1 mL portions of platelet-poor plasma were aliquoted into Eppendorf tubes and put on ice until plasma manipulation.

### 2.4. Plasma Manipulation

Plasma samples from math and recovery were subjected to the following four conditions: (a) not reconstituted or corrected, (b) corrected by the Dill and Costill equation, (c) reconstituted with baseline plasma, and (d) reconstituted with physiological saline. After each plasma manipulation, plasma samples were frozen at −80°C until assayed.

#### 2.4.1. Plasma Not Reconstituted or Corrected

Plasma was not manipulated and was analyzed for all coagulation measures.

#### 2.4.2. Plasma Corrected by the Dill and Costill Equation

From the values of coagulation parameters assessed in the uncorrected plasma, mathematical correction was made for PV shifts. The calculation for estimating PV changes incorporates both Hct (to determine the percentage of PV) and Hgb (to control for hemoconcentration-induced changes in red cell volume) before and after each manipulation [19]. The equation is

(1) $$\begin{array}{c} {\text{BV}}_{\text{A}}={\text{BV}}_{\text{B}}*\left(\frac{{\text{Hgb}}_{\text{B}}}{{\text{Hgb}}_{\text{A}}}\right),\\ {\text{CV}}_{\text{A}}={\text{BV}}_{\text{A}}*\left(\frac{{\text{Hct}}_{\text{A}}}{100}\right),\\ {\text{PV}}_{\text{A}}={\text{BV}}_{\text{A}}-{\text{CV}}_{\text{A}},\\ \%\Delta \text{PV}=100*\frac{\left({\text{PV}}_{\text{A}}-{\text{PV}}_{\text{B}}\right)}{{\text{PV}}_{\text{B}}}, \end{array}$$

where BV = blood volume, CV = red cell volume, PV = plasma volume, Hgb = hemoglobin, Hct = hematocrit, subscript B refers to baseline sample, subscript A refers to the period (math or recovery) sample, BV_B_ is taken as 100, and PV_B_ is 100 − Hct_B_. Given that changes in the actual size of red blood cells can affect the packed cell volume of Hct, Hgb is used in the PV equation to control for possible changes in mean corpuscular volume [22].

The corrected period (math, recovery) values for concentrations of coagulation measures were calculated from the measured levels during each period and the estimated percentage change in PV [18]. The equation is

(2) $$\begin{array}{c} C_{C}=\frac{C_{U}}{\left[1-\left(\%\Delta \text{PV}/100\right)\right]}, \end{array}$$

where *C*_*U*_ = measured coagulation parameter during each period.

#### 2.4.3. Plasma Reconstituted with Baseline Plasma

The 1 mL of plasma in each Eppendorf tube from math or recovery was reconstituted with an amount of plasma from baseline such that PV during math or recovery became equal to PV at baseline. This amount was determined from the following formula:

(3) $$\begin{array}{c} \mu \text{L}\;\text{per}\;1\;\text{mL}=\left[\left(\frac{{\text{PV}}_{\text{B}}}{{\text{PV}}_{\text{A}}}\right)*\left(\frac{{\text{CV}}_{\text{A}}}{{\text{CV}}_{\text{B}}}\right)-1\right], \end{array}$$

where PV_B_ is PV during baseline, PV_A_ is PV during math or recovery, CV_B_ is red cell volume during baseline, and CV_A_ is red cell volume during math or recovery. It was expected that PV would increase to above baseline levels during recovery in some individuals. If this occurred, plasma was not reconstituted.

#### 2.4.4. Plasma Reconstituted with Physiological Saline

Plasma samples from math and recovery were reconstituted via the same method as in the plasma reconstitution manipulation. However, instead of using the participant's own plasma from baseline, plasma was reconstituted with physiological saline (0.9% NaCl; Hospira, Inc., Lake Forest, IL, USA).

### 2.5. Hemoconcentration Measures

Hct and Hgb concentrations were determined in triplicate from EDTA tubes with a Coulter Counter (AcT 10). Hct was calculated from the red cell concentration, and Hgb concentration was determined by the cyanmethemoglobin method (Streck Mini-Pack, Omaha, NE, USA).

### 2.6. Hemostasis Measures

Fibrinogen, FVII:C, FVIII:C, APTT, and PT% were determined with the Beckman Coulter ACL 1000 by standard coagulometric methods using factor-deficient standard human plasma and reagents. D-dimer was determined using a microplate reader and an enzyme-linked immunosorbent assay (ZYMUTEST D-Dimer, HYPHEN BioMed, Neuville sur-Oise, France). Coefficients of variation for all hemostasis measures were less than 5%.

### 2.7. Statistical Analyses

Data were analyzed using SPSS (version 17.0, Chicago, IL, USA). All tests are two-tailed with level of significance set at *P* < .05. The Kolmogorov-Smirnov test was used to test for normality for hemoconcentration, hemostasis, and hemodynamic measures. The Huynh-Feldt correction was applied to account for any violations of the sphericity assumption for repeated measures, in which case the uncorrected degrees of freedom, the corrected *P* value, and the epsilon value are reported. Repeated measures ANOVAs were conducted for heart rate, DBP, SBP, Hct, and calculated PV with time (baseline, stress, and recovery) as the independent variable. Planned comparisons were used to compare stress (i.e., math) and recovery to baseline, using the Bonferroni adjustment for multiple comparisons (*α*_adj_ = .05/2 = .025). Based on previous findings [23], it was expected that recovery PV would not be significantly different from baseline PV. Therefore, if recovery plasma could not be reconstituted because calculated PV at recovery was greater than or equal to baseline PV, values equal to uncorrected recovery values were included for each reconstitution condition at recovery. Then, 3(Time) × 4(Plasma manipulation: uncorrected, arithmetic correction, saline reconstitution, plasma reconstitution) within-subjects ANOVAs were conducted to test for changes in each coagulation measure. Twenty planned comparisons were used to compare levels of hemostasis parameters after each plasma manipulation during stress and recovery to levels at baseline and to each other, using the Bonferroni adjustment for multiple comparisons (*α*_adj_ = .05/20 = .0025). To verify and complement this analysis, area-under-the-curve (AUC) with respect to the increase [21] was calculated for each coagulation measure after each plasma manipulation. For parameters whose AUC followed a normal distribution (i.e., FVII:C, FVIII:C, and PT%), a series of 4(Plasma manipulation) within-subjects ANOVAs were conducted with AUC for each coagulation parameter as the dependent variable. Planned comparisons were then used to compare AUC for coagulation measures across plasma manipulations, using the Bonferroni adjustment for multiple comparisons (*α*_adj_ = .05/6 = .0083). Marginal significance for planned comparisons is suggested at *α* < .05. AUC for fibrinogen, D-dimer, and APTT did not follow a normal distribution. Therefore, a series of Friedman's tests for repeated measures were conducted for these parameters, followed by Wilcoxon Signed-Rank posttests with the Bonferroni adjustment for multiple comparisons. Results are presented as mean ± SD and Cohen's *d* was calculated.

## 3. Results

### 3.1. Hemodynamic and Hemoconcentration Measures

Hemodynamic measurements were averaged at baseline, stress, and recovery. The sphericity assumption was violated for HR, SBP, and DBP. There were significant main effects of time for HR, *F*(2, 78) = 129.05, *ε* = .581, *P* < .001; SBP, *F*(2, 78) = 174.22, *ε* = .738, *P* < .001; DBP, *F*(2, 78) = 138.11, *ε* = .688, *P* < .001; hematocrit, *F*(2, 78) = 21.04, *ε* = 1.0, *P* < .001; and PV, *F*(2, 78) = 28.22, *ε* = 1.0, *P* < .001. SBP increased significantly from baseline (*M* = 122.19 ± 8.85) to poststress (*M* = 141.05 ± 11.8), *P* < .001, and remained elevated at recovery (*M* = 124.63 ± 9.06), *P* = .003. DBP increased significantly from baseline (*M* = 63.38 ± 6.87) to poststress (*M* = 76.43 ± 8.04), *P* < .001, and remained elevated at recovery (*M* = 64.97 ± 6.47), *P* = .021. HR increased significantly from baseline (*M* = 63.6 ± 10.31) to poststress (*M* = 81.03 ± 13.35), *P* < .001, and remained elevated at recovery (*M* = 64.84 ± 9.99), *P* = .029.

Hematocrit increased significantly from baseline (*M* = 42.26 ± 2.21) to stress (*M* = 43.17 ± 2.17), *P* < .001, but was not significantly different from baseline at recovery (*M* = 42.03 ± 1.98), *P* = .477. Plasma volume decreased significantly from baseline (*M* = 57.74 ± 2.21) to stress (*M* = 55.35 ± 2.67), *P* < .001, but was not significantly different from baseline at recovery (*M* = 58.17 ± 2.69), *P* = .771. The calculated reconstitution value was negative for eight participants (mean reconstitution amount = −25.78 *μ*L, range: −2.07 *μ*L to −61.63 *μ*L), despite a slight negative percent change in PV from baseline to stress in two participants (−0.36% and −0.71%). Thus, these participants were excluded from the reconstitution procedure.

### 3.2. Hemostasis Measures

#### 3.2.1. Factor VII Activity

**Figure 1:**
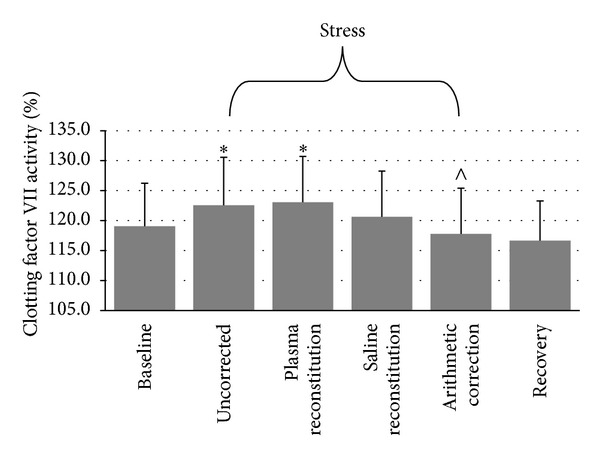
Differences in FVII:C across conditions (mean ± SEM). ∗: different from baseline at *P* < .02. ∧: different from uncorrected at *P* < .001.

Due to an insufficient amount of plasma, FVII:C was not assessed during stress for one participant, leaving 39 participants for this analysis. Repeated measures ANOVA indicated a significant time-by-plasma manipulation interaction, *F*(6, 228) = 7.2, *ε* = .564, *P* < .001. The nature of this interaction is shown in Figure 1. Repeated measures ANOVA indicated a significant main effect for FVII:C AUC, *F*(3, 144) = 5.08, *ε* = .807, *P* = .005. Uncorrected AUC (*M* = 21.03 ± 206.65) was significantly greater than AUC corrected arithmetically (*M* = −31.70 ± 225.74, *P* = .001) but not significantly different from AUC corrected with plasma (*M* = 32.43 ± 227.08, *P* = .467) or AUC corrected with saline (*M* = −6.38 ± 239.25, *P* = .165). AUC corrected with plasma was significantly greater than AUC corrected with saline (*P* = .005) and AUC corrected arithmetically (*P* = .004). AUC corrected with saline was not significantly different from AUC corrected arithmetically (*P* = .249). Estimated effect sizes for comparisons between levels of hemostasis parameters after each plasma manipulation during stress and recovery to levels at baseline and to each other are presented in Table 2, and estimated effect sizes for AUC comparisons are presented in Table 3.

#### 3.2.2. Factor VIII Activity

**Figure 2:**
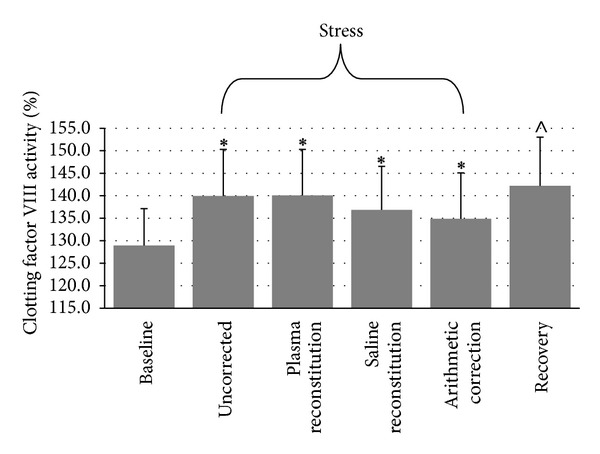
Differences in FVIII:C across conditions (mean ± SEM). ∗: different from baseline at *P* = .005. ∧: different from baseline at *P* = .02.

Repeated measures ANOVA indicated a nonsignificant time-by-plasma manipulation interaction, *F*(6, 234) = 2.13, *ε* = .488, *P* = .10, a nonsignificant main effect for plasma manipulation, *F*(1.9, 114.2) = 0.71, *P* = .49, and a significant main effect for time, *F*(1.6,114.2) = 4.06, *P* = .03. As shown in Figure 2, uncorrected FVIII:C significantly increased from baseline to stress (*P* = .005) and remained significantly elevated at recovery (*P* = .02), but plasma manipulation did not affect FVIII:C. Repeated measures ANOVA indicated no significant main effect for FVIII:C AUC, *F*(3, 117) = .933, *ε* = .632, *P* = .394. No significant differences emerged between uncorrected AUC (*M* = 275.83 ± 545.41), AUC corrected with plasma (*M* = 262.38 ± 477.35), AUC corrected with saline (*M* = 223.5 ± 494.28), or AUC corrected arithmetically (*M* = 224.48 ± 541.18).

#### 3.2.3. Prothrombin Time

**Figure 3:**
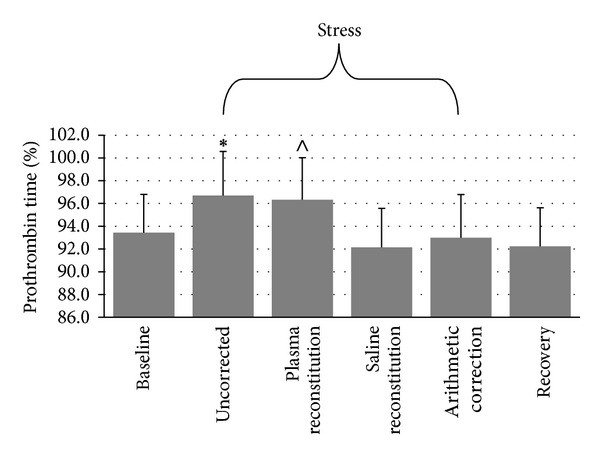
Differences in PT(%) across conditions (mean ± SEM). ∗: different from baseline, saline reconstitution, and arithmetic correction at *P* < .001. ∧: different from baseline at *P* = .012 and different from saline reconstitution and arithmetic correction at *P* < .001.

Repeated measures ANOVA indicated a significant time-by-plasma manipulation interaction, *F*(6, 234) = 7.51, *ε* = .564, *P* < .001, as depicted in Figure 3. Repeated measures ANOVA indicated a significant main effect for PT% AUC, *F*(3, 117) = 7.56, *ε* = .845, *P* < .001. Uncorrected AUC (*M* = 30.58 ± 116.36) was significantly greater than AUC corrected with saline (*M* = −33.33 ± 161.36, *P* = .002) and AUC corrected arithmetically (*M* = −9.07 ± 128.65, *P* = .001), but not significantly different from AUC corrected with plasma (*M* = 25.45 ± 131.35, *P* = .717). AUC corrected with plasma was significantly greater than AUC corrected with saline (*P* = .001) and marginally greater than AUC corrected arithmetically (*P* = .036). AUC corrected with saline was not significantly different from AUC corrected arithmetically (*P* = .148).

#### 3.2.4. Activated Partial Thromboplastin Time

**Figure 4:**
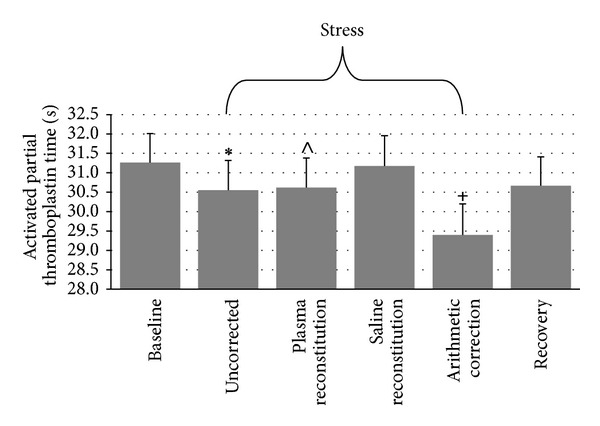
Differences in APTT across conditions (mean ± SEM). ∗: different from baseline and arithmetic correction at *P* < .001 and saline reconstitution at *P* = .006. ∧: different from baseline and saline reconstitution at *P* < .04. +: different from baseline, uncorrected, plasma reconstitution, and saline reconstitution at *P* < .001.

Figure 4 details the repeated measures ANOVA for APTT, which indicated a significant time-by-plasma manipulation interaction, *F*(6, 234) = 15.27, *ε* = .636, *P* < .001. Friedman's test was significant for APTT AUC, *X* ^2^(3) = 27.54, *P* < .001. Wilcoxon Signed-Rank tests suggest that uncorrected AUC (*M* = −15.21 ± 31.26) was significantly less than AUC corrected with saline (*M* = −6.31 ± 33.03, *P* < .001) and significantly greater than AUC corrected arithmetically (*M* = −26.67 ± 42.79, *P* = .001) but was not significantly different from AUC corrected with plasma (*M* = −13.48 ± 32.87, *P* = .35). AUC corrected with saline was significantly greater than AUC corrected arithmetically (*P* < .001) and significantly greater than AUC corrected with plasma (*P* = .005). AUC corrected with plasma was significantly greater than AUC corrected arithmetically (*P* = .004).

#### 3.2.5. Fibrinogen

**Figure 5:**
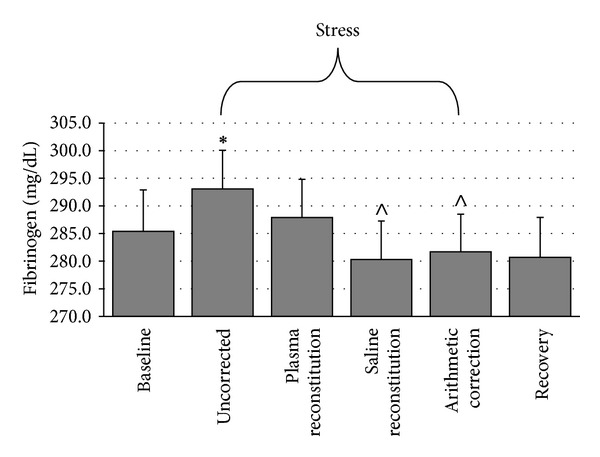
Differences in fibrinogen across conditions (mean ± SEM). ∗: different from baseline at *P* = .015. ∧: different from uncorrected at *P* < .001.

As shown in Figure 5, repeated measures ANOVA indicated a significant time-by-plasma manipulation interaction, *F*(6, 234) = 6.18, *ε* = .557, *P* < .001. Friedman's test was significant for fibrinogen AUC, *X* ^2^(3) = 18.80, *P* < .001. Wilcoxon Signed-Rank tests suggest that uncorrected AUC (*M* = 52.78 ± 469.23) was significantly greater than AUC corrected with saline (*M* = −110.23 ± 588.88, *P* < .001) and AUC corrected arithmetically (*M* = −67.03 ± 553.92, *P* = .001) but not significantly different from AUC corrected with plasma (*M* = 1.75 ± 636.12, *P* = .281). AUC corrected with plasma was marginally greater than AUC corrected with saline (*P* = .028). AUC corrected arithmetically was not significantly different from AUC corrected with plasma (*P* = .206) or AUC corrected with saline (*P* = .268).

#### 3.2.6. D-Dimer

**Figure 6:**
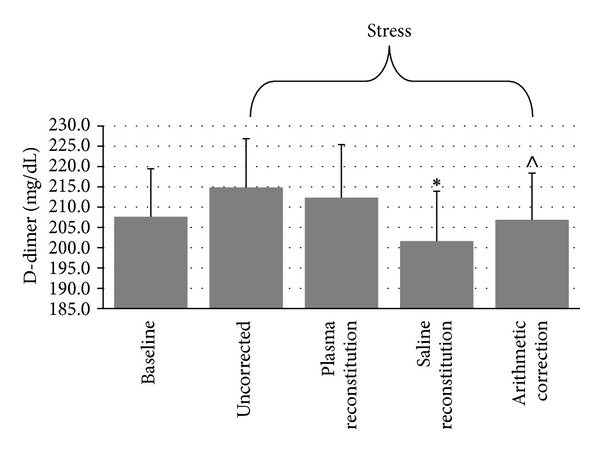
Differences in D-dimer across conditions (mean ± SEM). ∗: different from uncorrected and plasma reconstitution at *P* < .05. ∧: different from uncorrected at *P* < .001.

Due to budget restraints, plasma was analyzed for D-dimer only at baseline and stress in only 37 participants. Repeated measures ANOVA indicated a marginally significant time-by-plasma manipulation interaction, *F*(3, 108) = 2.93, *ε* = .617, *P* = .064, as observed in Figure 6. Given that D-dimer was only analyzed at baseline and stress, only 10 pairwise comparisons were made, with an adjusted significance level of *α*_adj_ = .005. AUC was calculated from baseline and stress values only. Friedman's test was significant for D-dimer AUC, *X* ^2^(3) = 16.28, *P* = .001. Wilcoxon Signed-Rank tests suggest that uncorrected AUC (*M* = 1289.23 ± 438.82) was significantly greater than AUC corrected arithmetically (*M* = 1241.25 ± 420.37, *P* < .001) and marginally greater than AUC corrected with saline (*M* = 1209.84 ± 448.58, *P* = .033). AUC corrected with plasma (*M* = 1274.22 ± 476.81) was marginally greater than AUC corrected with saline (*P* = .025). Other comparisons were nonsignificant (*P*s > .28).

## 4. Discussion

In this study, the mental arithmetic stressor evoked significant changes in hemodynamics, plasma volume, and coagulation. The results suggest heightened activity of the intrinsic pathway of the coagulation cascade (i.e., APTT and FVIII:C) during stress that remained elevated 20 minutes afterwards and transient increased activity of the extrinsic pathway of the coagulation cascade (i.e., PT% and FVII:C) during stress that returned to baseline levels following the termination of the stressor. Fibrinogen increased significantly from baseline to stress and returned to baseline levels at recovery, while D-dimer concentration did not increase significantly from baseline to stress. Altogether, the mental arithmetic stressor resulted in marked changes in coagulation. However, the main purpose of this investigation was to test the reconstitution methods against the usual arithmetic correction.

Fibrinogen levels were no longer significantly different from baseline levels after baseline plasma reconstitution, though levels were still somewhat elevated compared to uncorrected levels, suggesting that reconstitution with the person's own plasma from baseline incompletely reduced the fibrinogen concentration toward baseline levels. Though the changes in D-dimer from baseline to stress were nonsignificant for both uncorrected levels and for levels after baseline plasma reconstitution, they were in the expected direction and the pattern of results mirrored that of fibrinogen. Thus, it appears that plasma reconstitution incompletely removed the hemoconcentration effects on D-dimer and fibrinogen. After plasma reconstitution, the concentration of these substances was at a level between the baseline concentration and the uncorrected concentration but was not statistically different from either of them. Furthermore, the amount of baseline plasma used to reconstitute one milliliter of plasma from stress was typically very small (*M* = 55.4 *μ*L). Therefore, reconstitution with the person's own plasma from baseline did not have substantial effects on the concentrations of fibrinogen or D-dimer.

However, these results are somewhat at odds with the only previous study examining these reconstitution methods, in which we reported that D-dimer concentration after the Trier Social Stress Test (TSST) remained greater than the baseline concentration after baseline plasma reconstitution [14]. A possible explanation for this discrepancy is the use of different study protocols. In the current study, participants were tested at various times throughout the day and had a 20-minute baseline period, whereas participants in Austin et al.'s [14] were tested at the same time of the day and had a 120-minute baseline period. The baseline in our previous study may have been a truer baseline than the baseline in the current investigation. These differences in the protocol may have produced the slight differences in the relationships observed. On the other hand, like our previous investigation [14], uncorrected D-dimer concentration in this study was not significantly different from D-dimer concentration after correction with baseline plasma reconstitution.

On the other hand, APTT, PT%, FVII:C, and FVIII:C during stress were not altered by baseline plasma reconstitution, suggesting that reconstitution with the person's own plasma from baseline did not significantly alter the plasma's environment. These results make sense because more FVII, for example, is being introduced to the plasma collected during stress, but the FVII from baseline introduced to the stressor plasma has less activity than the FVII in the plasma collected during stress. However, this introduced FVII combined with the FVII in the stressor plasma does not appear to reduce the total activity of FVII. In other words, reconstitution with the person's own plasma from baseline did not alter FVII:C during stress and subsequently did not alter PT%. A similar line of reasoning can be made for FVIII:C and APTT. At recovery, fibrinogen, APTT, PT%, and FVII:C returned to baseline levels when uncorrected, and baseline plasma reconstitution did not alter their levels. Uncorrected FVIII:C remained elevated at recovery, but was not altered by baseline plasma reconstitution. Altogether, after baseline plasma reconstitution, APTT, PT%, and FVII:C remained elevated during stress and FVIII:C remained elevated during stress and during recovery, whereas fibrinogen and D-dimer were not significantly different from baseline. Based on the 3 × 4 repeated measures ANOVA, baseline plasma reconstitution removed hemoconcentration effects when examining concentrations (i.e., fibrinogen and D-dimer) but had no effect when examining clotting activity or clotting time. However, the results for fibrinogen and D-dimer were not confirmed by the AUC analyses, suggesting that the 3 × 4 ANOVA may have resulted in Type I error and that baseline plasma reconstitution did not influence any of the hemostasis parameters measured in this study.

Compared to the person's own plasma from baseline, we speculate that physiological saline is more likely to be representative of the filtrate that is lost through capillary pores during acute stress, as this filtrate is free of large, nondiffusible molecules. It was hypothesized that, when reconstituted with physiological saline, plasma obtained during an acute math stressor or during recovery would no longer have different fibrinogen, D-dimer, APTT, PT%, or FVII:C than plasma obtained during baseline but would still have elevated FVIII:C than plasma obtained during baseline. As predicted, fibrinogen was no longer significantly different from baseline levels after saline reconstitution. Similar to the baseline plasma reconstitution, the change in D-dimer from baseline to stress was nonsignificant after saline reconstitution, but was in the expected direction and mirrored the change in fibrinogen. These results are expected, given that saline reconstitution dilutes the stressor plasma in such a way that the concentrations of fibrinogen and D-dimer should be equivalent to baseline levels. These results corroborate our previous work [14], which showed that saline reconstitution after the TSST resulted in fibrinogen and D-dimer being no longer significantly different from baseline.

Like fibrinogen and D-dimer, when reconstituted with saline reconstitution after stress, APTT, PT%, and FVII:C were not significantly different than at baseline. These results suggest that saline reconstitution reduced clotting time (APTT and PT%) and FVII:C. When plasma becomes more diluted, clotting factors have less opportunity to interact with other clotting factors and with the endothelium, thereby reducing clotting activity. In effect, hemoconcentration seems to have been responsible for the increase in FVII:C and decrease in clotting time. On the other hand, FVIII:C remained elevated after saline reconstitution, suggesting actual activation of FVIII independent of hemoconcentration. This result was predicted and is in line with previous research [14, 15, 24] that has suggested genuine activation of the intrinsic pathway of the coagulation system during acute stress. At recovery, fibrinogen, APTT, PT%, and FVII:C returned to baseline levels when uncorrected, and saline reconstitution did not alter their levels. When uncorrected, FVIII:C remained elevated at recovery, and saline reconstitution did not alter FVIII:C. Altogether, saline reconstitution removed hemoconcentration effects when examining concentrations, clotting time of the intrinsic and extrinsic pathways, and clotting activity of the extrinsic pathway but had no effect when examining clotting activity of the intrinsic pathway. The results for APTT and FVIII:C are puzzling because if there was complete activation of the intrinsic pathway, then APTT at stress should have survived correction with saline reconstitution. Nevertheless, one must bear in mind that previous studies [14, 15, 24] suggested that FVIII:C is genuinely activated, but not necessarily APTT. Contrary to baseline plasma reconstitution, AUC analyses confirmed the findings of the 3 × 4 ANOVA, pointing to the robustness of the saline reconstitution technique.

Arithmetic correction appears to be appropriate when correcting for the effects of PV shifts on concentrations of large, non-diffusible molecules but seems to improperly adjust when examining clotting time and clotting activity, as previously suggested [14]. When examining concentrations in the current investigation, arithmetic correction removed stress effects of fibrinogen and D-dimer. During stress, fibrinogen corrected arithmetically was marginally less than fibrinogen corrected with baseline plasma reconstitution and not significantly different than fibrinogen corrected with saline reconstitution. Again, the pattern of results for D-dimer was similar to that of fibrinogen. Altogether, either arithmetic correction or saline reconstitution appears to be an appropriate technique of adjusting for hemoconcentration effects on fibrinogen and D-dimer. It is likely that arithmetic correction and correction with saline reconstitution are also appropriate and correction with baseline plasma reconstitution is inappropriate for other large, non-diffusible molecules (e.g., lipids, plasma proteins, or other clotting factors).

The significant effects of stress on FVII:C were removed after arithmetic correction and after correction with saline reconstitution. In spite of this, closer examination of the data suggests that saline reconstitution only partially removed hemoconcentration effects, whereas only arithmetic correction completely eliminated hemoconcentration effects. Given the findings for fibrinogen, D-dimer, and FVIII:C, however, one would expect saline reconstitution and arithmetic correction to exhibit equivalent correction effects, as saline reconstitution is more or less the physical equivalent of arithmetic correction. Based on these findings, it is unclear which PV correction technique is most appropriate for FVII:C.

During stress and recovery, no correction technique completely removed hemoconcentration effects on FVIII:C, suggesting that FVIII:C increases during stress independent of hemoconcentration. Thus, in line with previous studies [14, 15, 24], the intrinsic pathway of the coagulation system seems to be truly activated during acute psychological stress.

When examining clotting time, both arithmetic correction and saline reconstitution removed the effects of hemoconcentration on PT%. However, the arithmetically corrected finding is at odds with our previous studies [14, 15], which showed that arithmetically adjusted PT% was less than PT% at baseline, suggesting that correction for hemoconcentration resulted in slower clotting time of the extrinsic pathway. We [14] previously suggested that arithmetic correction overcorrected for changes in PT% that are associated with PV shifts. It is not entirely clear, however, why the Dill and Costill [19] formula did not overcorrect in the current study. Conversely, arithmetic correction appeared to improperly adjust for APTT in the current study. Similar to previous findings [14, 15], instead of APTT being adjusted back toward its baseline level, it decreased, suggesting that clotting speed becomes faster when taking into account PV shifts. However, this finding is incommensurate with the finding that FVIII:C decreases slightly and fibrinogen is no longer different from baseline after arithmetic adjustment. One would expect a decrease in FVIII:C and fibrinogen to be accompanied by a prolongation in APTT. In other words, a reduction in clotting activity or concentration should be associated with a slower clotting response. The Dill and Costill [19] formula is designed to mathematically adjust the concentration of a physiological parameter based on changes in PV but appears to be of limited utility when correcting for PV changes on time-dependent functional assays such as clotting time. Finally, the AUC analyses confirmed the results observed for arithmetic correction.

Several unmeasured physiological parameters could have played a role in the observed changes in PV and coagulation. For example, changes in colloid osmotic pressure parallel changes in hematocrit and arterial pressure and may act as an underlying mechanism of stress hemoconcentration, especially during recovery [6, 7]. Nuclear factor kappa B (NF-*κ*B) is a proinflammatory, procoagulant genetic transcription factor that is activated by increased shear stress [25]. Acute psychological stress increases shear stress, thereby stimulating NF-*κ*B activation. Therefore, NF-*κ*B activation could be a key mediator between stress, elevated coagulation activity, and atherogenesis [26, 27]. Given that stress hemoconcentration is associated with greater shear stress [5], interrelationships between colloid osmotic pressure, NF-*κ*B, coagulation, and hemoconcentration during stress should be examined. On the other hand, it is unlikely that proinflammatory cytokines such as interleukin-6 contributed to the stress response of our coagulation measures, because inflammatory responses to acute psychosocial stress are typically delayed up to 60 minutes after termination of a stressor [28].

### 4.1. Limitations and Directions for Future Research

Limitations of the present study must be acknowledged. First, due to lack of funding, D-dimer was not measured at recovery. However, given that we [14] have reported that D-dimer returned to baseline levels and that no PV correction had any effect on D-dimer at recovery and given that reconstitution had no effect on any parameter at recovery in the current investigation, it is expected that D-dimer would have returned to baseline levels and that reconstitution would not have influenced its concentration. Second, given the very small amounts of plasma and saline used in the reconstitution procedure, pipetting errors were possible, potentially skewing results. Finally, the results of the current study cannot be generalized to women or individuals with disease, as the study sample consisted solely of university students. This is especially important given that hypercoagulability may be particularly harmful in individuals with developed coronary heart disease. Acute stress could promote atherogenesis through contracted PV and subsequent exposure of the endothelium to clotting activity. Future research should attempt to determine if hemoconcentration and coagulation reactivity confers risk for development of atherothrombotic disease and for future coronary events.

## 5. Conclusions

Two novel reconstitution methods for correcting hemoconcentration effects on stress-induced coagulation changes were examined in this study. Both the saline and baseline plasma reconstitution methods appeared to adequately adjust for PV shifts when examining concentrations, but the baseline plasma reconstitution method had inconsequential effects on clotting time and clotting activity. Thus, the baseline plasma reconstitution method does not appear to be an informative hemoconcentration correction technique, because the Dill and Costill [19] formula adjusts equally as well or better without having the extra step in the laboratory of reconstitution. The saline reconstitution method, on the other hand, may be the most biologically relevant correction technique when examining stress-hemoconcentration effects on clotting time and clotting activity, whereas the Dill and Costill formula does not seem appropriate. With the exception of FVIII:C, hemoconcentration appears to account for most of the stress-induced changes in the coagulation parameters examined in this study. The intrinsic pathway of the coagulation system, however, is most likely genuinely activated during acute stress, as indicated by increases in FVIII:C surviving all hemoconcentration correction techniques. Altogether, stress-induced changes in coagulation are a consequence of both hemoconcentration and actual activation of the coagulation system.

## Figures and Tables

**Table 1:** Participant characteristics, *N* = 40.

Characteristics (mean ± SD)	
Age (years)	19.85 ± 2.56
BMI (kg/m^2^)	24.02 ± 2.94
Screening SBP (mmHg)	114.38 ± 8.41
Screening DBP (mmHg)	74.11 ± 9.26
Carbon monoxide (parts per million)	1.71 ± 1.60
Blood alcohol content (%)	0.00 ± 0.00

**Table 2:** Estimated effect sizes for comparisons of levels of hemostasis measures after each plasma manipulation during stress and recovery to levels at baseline and to each other.

Comparison	Cohen's *d *					
	Fibrinogen	D-Dimer	FVIII:C	FVII:C	APTT	PT%
Baseline-Stress_U_	0.17	0.10	0.19	0.08	0.15	0.15
Baseline-Stress_B_	0.05	0.06	0.19	0.09	0.13	0.13
Baseline-Stress_S_	0.11	0.08	0.14	0.04	0.02	0.06
Baseline-Stress_A_	0.08	0.01	0.10	0.03	0.38	0.02
Stress_U_-Stress_B_	0.12	0.03	0.02	0.01	0.02	0.02
Stress_U_-Stress_S_	0.29	0.18	0.05	0.03	0.13	0.20
Stress_U_-Stress_A_	0.26	0.11	0.08	0.10	0.23	0.15
Stress_B_-Stress_S_	0.18	0.14	0.05	0.05	0.11	0.19
Stress_B_-Stress_A_	0.14	0.08	0.08	0.11	0.25	0.14
Stress_S_-Stress_A_	0.03	0.08	0.03	0.07	0.36	0.04
Baseline-Recovery_U_	0.10	N/A	0.22	0.05	0.13	0.06
Baseline-Recovery_B_	0.07	N/A	0.20	0.04	0.11	0.06
Baseline-Recovery_S_	0.09	N/A	0.21	0.07	0.11	0.08
Baseline-Recovery_A_	0.01	N/A	0.24	0.04	0.05	0.02

Subscript U refers to uncorrected values. Subscript B refers to baseline plasma reconstitution values. Subscript S refers to saline reconstitution values. Subscript A refers to arithmetically corrected values.

**Table 3:** Estimated effect sizes for area-under-the-curve.

Comparison	Cohen's *d *					
	Fibrinogen	D-dimer	FVIII:C	FVII:C	APTT	PT%
Uncorrected-plasma	0.09	0.03	0.03	0.05	0.06	0.04
Uncorrected-saline	0.31	0.18	0.10	0.12	0.28	0.45
Uncorrected-arithmetic	0.23	0.11	0.09	0.24	0.31	0.32
Plasma-saline	0.18	0.14	0.08	0.17	0.22	0.40
Plasma-arithmetic	0.12	0.07	0.07	0.28	0.35	0.26
Saline-arithmetic	0.08	0.08	0.001	0.11	0.53	0.17

## References

[1] Kung HC, Hoyert DL, Xu J, Murphy SL (2008). Deaths: final data for 2005. *National Vital Statistics Reports*.

[2] Greenland P, Knoll MD, Stamler J (2003). Major risk factors as antecedents of fatal and nonfatal coronary heart disease events. *Journal of the American Medical Association*.

[3] Rosengren A, Hawken S, Ôunpuu S (2004). Association of psychosocial risk factors with risk of acute myocardial infarction in 11119 cases and 13648 controls from 52 countries (the INTERHEART study): case-control study. *The Lancet*.

[4] Strike PC, Steptoe A (2005). Behavioral and emotional triggers of acute coronary syndromes: a systematic review and critique. *Psychosomatic Medicine*.

[5] Alien MT, Patterson SM (1995). Hemoconcentration and stress: a review of physiological mechanisms and relevance for cardiovascular disease risk. *Biological Psychology*.

[6] de Boer D, Ring C, Curlett AC, Ridley M, Carroll D (2007). Mental stress-induced hemoconcentration and its recovery: a controlled study of time course and mechanisms. *Psychophysiology*.

[7] de Boer D, Ring C, Wood M (2007). Time course and mechanisms of mental stress-induced changes and their recovery: hematocrit, colloid osmotic pressure, whole blood viscosity, coagulation times, and hemodynamic activity. *Psychophysiology*.

[8] Hathcock JJ (2006). Flow effects on coagulation and thrombosis. *Arteriosclerosis, Thrombosis, and Vascular Biology*.

[9] Bhattacharyya MR, Steptoe A (2007). Emotional triggers of acute coronary syndromes: strength of evidence, biological processes, and clinical implications. *Progress in Cardiovascular Diseases*.

[10] Rozanski A, Blumenthal JA, Kaplan J (1999). Impact of psychological factors on the pathogenesis of cardiovascular disease and implications for therapy. *Circulation*.

[11] von Känel R, Mills PJ, Fainman C, Dimsdale JE (2001). Effects of psychological stress and psychiatric disorders on blood coagulation and fibrinolysis: a biobehavioral pathway to coronary artery disease?. *Psychosomatic Medicine*.

[12] Austin AW, Patterson SM, von Känel R (2011). Hemoconcentration and hemostasis during acute stress: interacting and independent effects. *Annals of Behavioral Medicine*.

[13] Thrall G, Lane D, Carroll D, Lip GYH (2007). A systematic review of the effects of acute psychological stress and physical activity on haemorheology, coagulation, fibrinolysis and platelet reactivity: implications for the pathogenesis of acute coronary syndromes. *Thrombosis Research*.

[14] Austin AW, Wirtz PH, Patterson SM, Stutz M, von Känel R (2012). Stress-induced alterations in coagulation: assessment of a new hemoconcentration correction technique. *Psychosomatic Medicine*.

[15] von Känel R, Kudielka BM, Haeberli A, Stutz M, Fischer JE, Patterson SM (2009). Prothrombotic changes with acute psychological stress: combined effect of hemoconcentration and genuine coagulation activation. *Thrombosis Research*.

[16] Zgraggen L, Fischer JE, Mischler K, Preckel D, Kudielka BM, von Känel R (2005). Relationship between hemoconcentration and blood coagulation responses to acute mental stress. *Thrombosis Research*.

[17] Steptoe A, Kunz-Ebrecht S, Owen N (2003). Influence of socioeconomic status and job control on plasma fibrinogen responses to acute mental stress. *Psychosomatic Medicine*.

[18] Muldoon MF, Bachen EA, Manuck SB, Waldstein SR, Bricker PL, Bennett JA (1992). Acute cholesterol responses to mental stress and change in posture. *Archives of Internal Medicine*.

[19] Dill DB, Costill DL (1974). Calculation of percentage changes in volumes of blood, plasma, and red cells in dehydration. *Journal of Applied Physiology*.

[20] Darlington DN, Delgado AV, Kheirabadi BS (2011). Effect of hemodilution on coagulation and recombinant factor VIIa efficacy in human blood in vitro. *The Journal of Trauma*.

[21] Pruessner JC, Kirschbaum C, Meinlschmid G, Hellhammer DH (2003). Two formulas for computation of the area under the curve represent measures of total hormone concentration versus time-dependent change. *Psychoneuroendocrinology*.

[22] van Beaumont W (1972). Evaluation of hemoconcentration from hematocrit measurements. *Journal of Applied Physiology*.

[23] Patterson SM, Krantz DS, Jochum S (1995). Time course and mechanisms of decreased plasma volume during acute psychological stress and postural change in humans. *Psychophysiology*.

[24] von Känel R, Preckel D, Zgraggen L (2004). The effect of natural habituation on coagulation responses to acute mental stress and recovery in men. *Thrombosis and Haemostasis*.

[25] Boon RA, Horrevoers AJG (2009). Key transcriptional regulators of the vasoprotective effects of shear stress. *Hamostaseologie*.

[26] Bierhaus A, Wolf J, Andrassy M (2003). A mechanism converting psychosocial stress into mononuclear cell activation. *Proceedings of the National Academy of Sciences of the United States of America*.

[27] Bierhaus A, Humpert PM, Nawroth PP (2004). NF-*κ*B as a molecular link between psychosocial stress and organ dysfunction. *Pediatric Nephrology*.

[28] Steptoe A, Hamer M, Chida Y (2007). The effects of acute psychological stress on circulating inflammatory factors in humans: a review and meta-analysis. *Brain, Behavior, and Immunity*.

